# Supplementary material for: Optimized cell systems for the investigation of hepatitis C virus E1E2 glycoproteins
Source: J Gen Virol. 2020 Nov 4;102(1):jgv001512. doi: 10.1099/jgv.0.001512 (PMC8116788; doi:10.1099/jgv.0.001512)
Supplement: Supplementary material 1 [file jgv-102-512-s001.pdf]

Supporting Information for

Optimised cell systems for the investigation of HCV E1E2 glycoproteins

Kalemera, Capella-Pujol, Chumbe et. al.

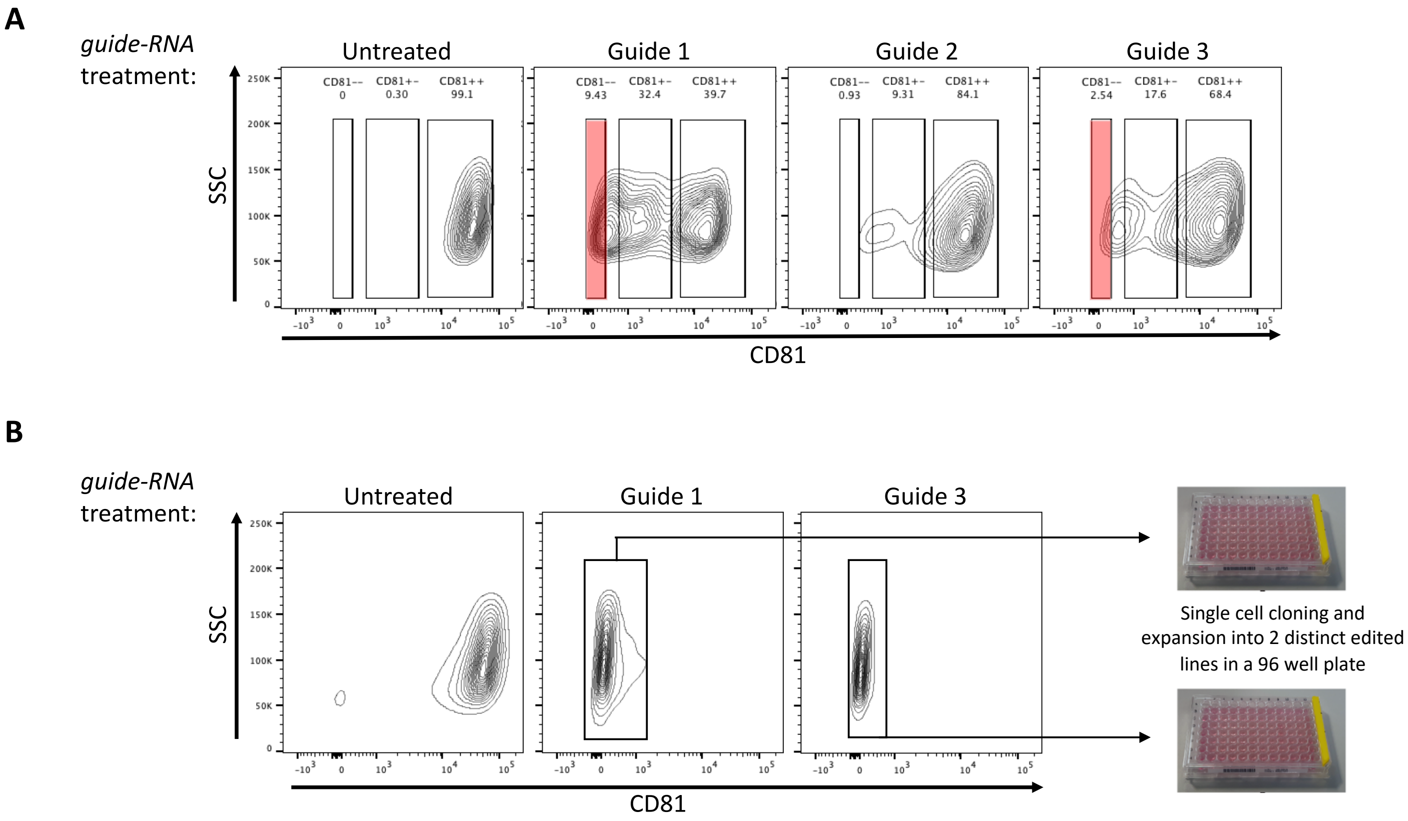

Supplementary Figure 1. Generation of CD81 knock-out 293T cell lines. 293T cells were transfected with CRISPR Cas9 gene-editing components along with one of three guide RNAs (sgRNA) targeting the CD81 exon. **(A)** 72 hours later cells were sorted using flow cytometry to obtain CD81 double negative populations (CD81-/-) (red coloured boxes). **(B)** Cells that received sgRNAs guide 1 and 3 were then expanded for 48 hours before dilution for single cell cloning and expansion in a 96 well plate.

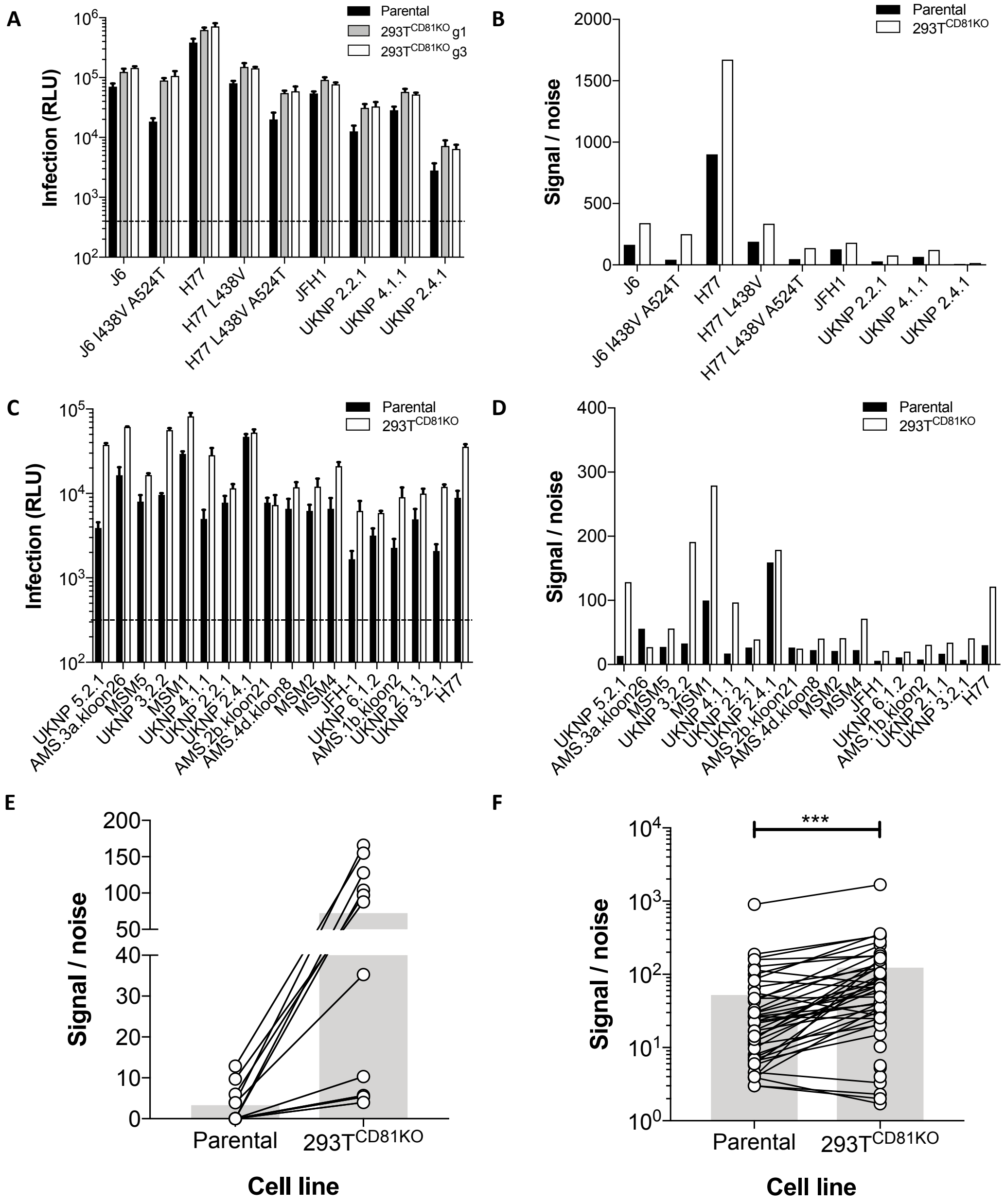

Supplementary Figure 2. HCVpp made in CD81 knock-out 293T cells exhibit enhanced infectivity. Huh-7 cells were infected with HCVpp produced in parental or 293T^{CD81KO} cell lines. The infection levels **(A)** and calculated S/N **(B)** for an HCVpp panel consisting of both prototypical and clinical isolates. Note, only S/N of virus made in 293T^{CD81KO} g3 cells is included in b. The infection levels **(C)** and calculated S/N **(D)** of a clinical isolate HCVpp panel. Error bars in a and c indicate the standard deviations between three replicate wells. **(E)** Stratification of the top improving clones and clones whose infection was rescued when produced in 293T^{CD81KO} cells. **(F)** A compiled summary of the calculated S/N for all screened clones (see Table S1). Paired t-test, (***p < 0.001). Connected points indicate a single E1E2 clone and the grey bars represent the mean value. All data are from lone experiments performed in triplicate. Dash line indicates background signal determined by infection of pseudoparticle lacking E1E2.

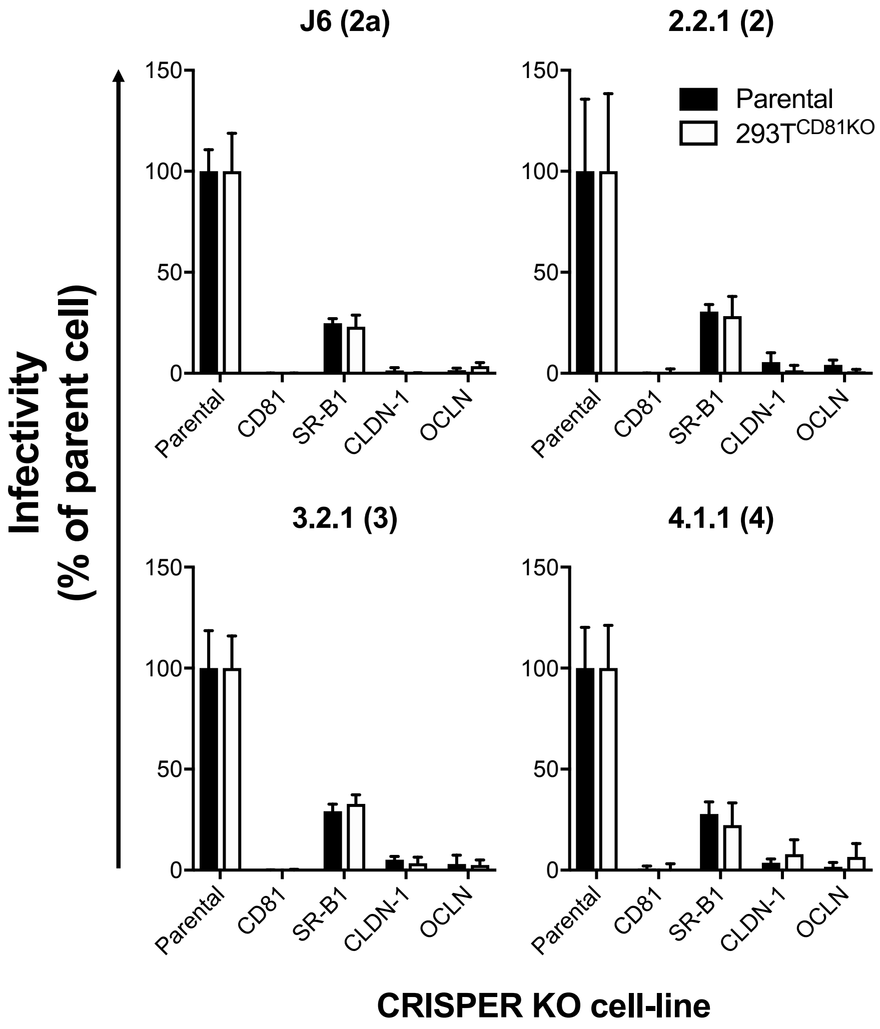

Supplementary Figure 3. Receptor dependency of 293T^{CD81KO} produced HCVpp. Huh-7 cell lines CRISPR/Cas9 engineered to not express one HCV receptor were infected with HCVpp produced in parental or 293T^{CD81KO} cell lines. Data are expressed as a percentage of the infection observed in parental Huh-7 cells. Error bars indicate the standard deviations between replicate wells. Data are representative of two independent experiments performed in quadruplicate.

HCVpp [a]	Core [b]	Genotype	293T line of HCVpp production		Fold change in S/N [c]
			Parental	$293T^{CD81KO}$	
H77	Lentiviral	1a	901.34	1673.03	1.86
H77 L438V ✤	Lentiviral	1a	188.55	335.28	1.78
H77 L438V A524T ✤	Lentiviral	1a	47.04	138.06	2.94
J6	Lentiviral	2a	164.88	340.21	2.06
J6 I438V A524T ◆	Lentiviral	2a	43.05	250.15	5.81
JFH1	Lentiviral	2a	127.48	181.22	1.42
UKNP 2.2.1	Lentiviral	2	29.57	77.21	2.61
UKNP 2.4.1	Lentiviral	2	6.57	15.02	2.29
UKNP 4.1.1	Lentiviral	4	66.53	122.53	1.84
AMS.1b.kloon2	Retroviral	1b	7.96	30.86	3.88
AMS.2b.kloon21	Retroviral	2b	26.45	24.91	0.94
AMS.3a.kloon26	Retroviral	3a	55.90	27.27	0.49
AMS.4d.kloon8	Retroviral	4d	22.47	40.43	1.80
h77	Retroviral	1a	30.11	121.48	4.03
jfh1	Retroviral	2a	5.79	21.29	3.68
msm1	Retroviral	1	99.77	279.03	2.80
msm2	Retroviral	2	21.22	41.13	1.94
msm4	Retroviral	4	22.46	71.53	3.19
msm5	Retroviral	5	27.34	56.30	2.06
uknp 2.1.1	Retroviral	3	26.61	39.23	1.47
uknp 2.2.1	Retroviral	2	159.01	178.74	1.12
uknp 2.4.1	Retroviral	2	16.94	34.02	2.01
uknp 3.2.1	Retroviral	3	7.30	40.99	5.62
uknp 3.2.2	Retroviral	3	32.75	191.18	5.84
uknp 4.1.1	Retroviral	4	17.11	96.81	5.66
uknp 5.2.1	Retroviral	5	13.38	128.37	9.59
uknp 6.1.2	Retroviral	6	11.04	20.17	1.83
168_Cl T/F	Retroviral	1b	85.14	360.00	4.23
277_Cl T/F	Retroviral	3a	4.60	3.30	0.72
360_Cl T/F	Retroviral	1a/2b	3.90	35.30	9.05
686_Cl T/F	Retroviral	1a	6.90	25.50	3.70
4032_Cl T/F	Retroviral	3a	3.90	1.70	0.44
4087_Cl T/F	Retroviral	1b	0.00	128.00	128.00
023_Ch T/F1	Retroviral	1a	12.13	64.50	5.32
023_Ch T/F2	Retroviral	1a	0.00	10.30	10.30
023_197DPI	Retroviral	1a	0.00	5.30	5.30
240_Ch T/F	Retroviral	3a	3.00	2.30	0.77
256_Ch V1	Retroviral	1a	9.70	88.00	9.07
256_Ch V2	Retroviral	3a	6.00	97.00	16.17
256_79DPI 1	Retroviral	3a	0.00	4.00	4.00
256_287DPI	Retroviral	3a	3.00	2.00	0.67
HOK_Ch T/F	Retroviral	1b	12.90	155.00	12.02
HOK_30DPI	Retroviral	1b	4.00	166.00	41.50
HOK_233DPI	Retroviral	1b	0.00	5.70	5.70
THD T/F	Retroviral	1a	14.30	71.30	4.99
THD_109DPI	Retroviral	1a	114.30	74.50	0.65
THD_198DPI	Retroviral	1a	0.00	104.00	104.00
THG_Ch T/F	Retroviral	1a	14.30	71.30	4.99
THG_58DPI	Retroviral	1a	82.50	64.80	0.79
THG_184DPI	Retroviral	1a	47.00	49.00	1.04

Supplementary Table 1. Signal-to-noise ratio (S/N) for all HCV strains tested in the study. [a] E1E2 identification, [b] gag-pol genes origins: Lentiviral (HIV) and Retroviral (MLV), [c] fold change in HCVpp S/N (relative to S/N of parental 293T produced HCVpp), T/F: Transmission founder, ✤ E1E2 sequence of H77 mutants generated via site-directed mutagenesis, ◆ E1E2 sequence of an unpublished full-length cell-culture adapted J6/JFH chimeric strain.
